# Total synthesis of (±)-coerulescine and (±)-horsfiline

**DOI:** 10.3762/bjoc.6.103

**Published:** 2010-09-27

**Authors:** Mukund G Kulkarni, Attrimuni P Dhondge, Sanjay W Chavhan, Ajit S Borhade, Yunnus B Shaikh, Deekshaputra R Birhade, Mayur P Desai, Nagorao R Dhatrak

**Affiliations:** 1 Department of Chemistry, University of Pune, Ganeshkhind, Pune-411 007, Maharashtra, India

**Keywords:** alkaloids, Claisen rearrangement, Jones oxidation, spiro-oxindole, Wittig olefination

## Abstract

Wittig olefination–Claisen rearrangement protocol was applied to obtain 3-allyl oxindole. This oxindole was then converted to (±)-coerulescine and (±)-horsfiline.

## Introduction

**Figure 1:**
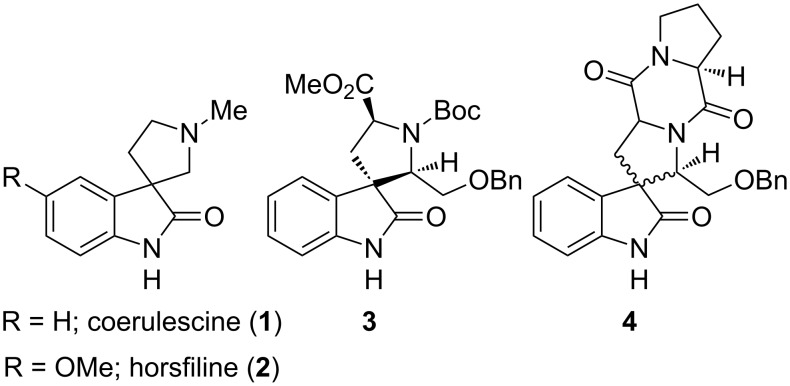
Spiro[pyrrolidin-3,3'-oxindoles].

The spiro[pyrrolidin-3,3′-oxindole] ring system is a widely distributed structural framework present in a number of cytostatic alkaloids. For example, coerulescine (**1**) and horsfiline (**2**) represent the simplest prototype members of this subfamily. Coerulescine (**1**) was isolated from the blue canary grass, *Phalaris coerulescens* [1–2]. Horsfiline (**2**) was first isolated in 1991 by Bodo from a Malaysian medical plant, *Horsfieldia superba* Warb [3]. Several Myristicaceae are used as a source of intoxicating snuffs [1–3]. Other members of this subfamily, such as spirotryprostatins A and B [4–5], elacomine [6] and rychnophylline [7–8], have more complex structures. The majority of these alkaloids have interesting biological activities and pharmacological properties [9]. However, a crucial observation, reported by Danishefsky et al. [10], found that the unnatural analogous **3** and **4** (Figure 1) of the spiro[pyrrolidin-3,3′-oxindole] possessed significant activity against human breast cancer cells. This work led to intense interest in the total synthesis of these alkaloids and their derivatives. Despite previous intensive studies, the total synthesis of coerulescine (**1**) and horsfiline (**2**) remain attractive targets for demonstrating the efficacy of newer synthetic protocols.

Several synthetic approaches have been developed for the synthesis of the spiro[pyrrolidin-3,3'-oxindole] framework for horsfiline and coerulescine [11–34], both in racemic and enantiomeric forms. These include the following oxidative rearrangements: lead tetraacetate [3], sodium tungstate [11], tert-butyl hypochlorite [12] and *N*-bromosuccinimide [13]. Other approaches involve the Mannich reaction [14], ring expansion reactions [15–16], 1,3-dipolar [3 + 2] cycloadditions [17–19], intramolecular radical cyclizations [20–24], electrophilic cyclization [25], asymmetric nitroolefination reaction [26], palladium asymmetric allylic alkylation [27], palladium-catalyzed domino Heck–cyanation [28], Pd-catalyzed intramolecular cyanoamidation [29–30], NHC-mediated O- to C-carboxyl transfer [31], dimethyldioxirane (DMDO) mediated oxidation [32], and by tandem intramolecular photocycloaddition–retro-Mannich reaction [33].

**Scheme 1:**
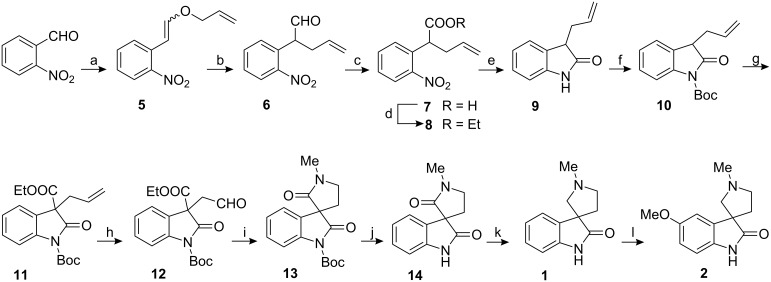
Reagents and conditions: a) CH_2_=CHCH_2_OCH_2_P^+^ Ph_3_Cl^−^, *t*-BuO^−^ Na^+^, THF, 0 °C; b) xylene, reflux; c) Jones reagent, acetone, d) H_2_SO_4_, EtOH, e) Zn, NH_4_Cl, EtOH, reflux, f) NaH, (Boc)_2_O, THF, 0 °C, g) NaH, ethyl chloroformate, THF, 0 °C, h) K_2_OsO_4_, NMO, NaIO_4_·SiO_2_, DCM, i) MeNH_2_·HCl, NaCNBH_3_, THF j) 2.5 M HCl aq. THF, reflux; k) *n-*BuLi, LAH, THF, l) NBS, NaOMe, CuI, reflux.

The Wittig olefination–Claisen rearrangement protocol [35] provides a ready access to 4-pentenals, which have served as versatile intermediates for the synthesis of a number of natural products [36–44]. Therefore, we describe the successful application of the above protocol for the synthesis of the coerulescine (**1**) and horsfiline (**2**) (Scheme 1).

## Results and Discussion

The Wittig olefination of *o*-nitrobenzaldehyde with allyloxymethylenetriphenylphosphorane under standard conditions [35] furnished the corresponding allyl vinyl ether **5** as an inseparable mixture of *E* and *Z* isomers. However, the NMR signals of the *E* and *Z* isomers in the olefinic region were well separated, which allowed us to estimate the ratio of these isomers as 1:2. The mixture of allyl vinyl ethers was heated in refluxing xylene to effect the Claisen rearrangement to obtain 4-pentenal **6** in 85% yield. Aldehyde **6** was transformed into acid **7** by Jones oxidation, which was immediately converted to the ethyl ester **8**. Subsequently, reduction of the compound **8** with Zn and NH_4_Cl resulted in clean cyclization leading to oxindole **9**.

After protecting the amide nitrogen with Boc, the oxindole **10** was treated with NaH, followed by ethyl chloroformate at 0 °C to give **11** in 80% yield. Oxidative cleavage of the allyl group was accomplished by catalytic osmium tetroxide and *N*-methylmorpholine *N*-oxide (NMO), followed by cleavage of the diol with sodium metaperiodate on silica in methylene chloride. Reductive amination of the aldehyde **12** was conducted using methylamine hydrochloride and NaBH_3_CN and gave spiro-oxindole **13**. The Boc group of **13** was removed by treatment with 2.5 M HCl to give **14**. Finally, chemoselective reduction of amide **14** with *n-*BuLi and LAH (under the conditions reported in [27]) gave coerulescine. Compound **1**, on treatment with *N*-bromosuccinimide, gave the 5-bromo derivative, which upon heating with sodium methoxide in the presence of cuprous iodide gave horsfiline in 60% yield. The physical data of synthetic coerulescine and horsfiline were comparable in all respects with the literature data.

## Conclusion

In summary, we have described a new and efficient synthesis of (±)-coerulescine and (±)-horsfiline.

## Supporting Information

File 1 Experimental and spectral data.
