# Supplementary material for: Total synthesis of (±)-coerulescine and (±)-horsfiline
Source: Beilstein J Org Chem. 2010 Sep 27;6:876–9. doi: 10.3762/bjoc.6.103 (PMC2982140; doi:10.3762/bjoc.6.103)
Supplement: File 1 — Experimental and spectral data. [file Beilstein_J_Org_Chem-06-876-s001.pdf]

Supporting Information

for

Total synthesis of (±)-coerulescine and (±)-horsfiline

Mukund G. Kulkarni*, Attrimuni P. Dhondge, Sanjay W. Chavhan, Ajit S. Borhade, Yunnus B. Shaikh, Deekshaputra R. Birhade, Mayur P. Desai and Nagorao R. Dhatrak

Address: Department of Chemistry, University of Pune, Ganeshkhind, Pune-411 007, Maharashtra, India

Email: Mukund G. Kulkarni - mgkulkarni@chem.unipune.ernet.in

* Corresponding author

Experimental and spectral data

1-(2-Allyloxy-1-vinyl)-2-nitrobenzene (5) A solution of *t*-$BuO^{-}Na^{+}$ (3.8 g, 0.039 mol) in dry THF was added dropwise to a suspension of *o*-nitrobenzaldehyde (5 g, 0.033 mol) and allyloxymethylenetriphenylphosphonium chloride [35] (14.6 g, 0.039 mol) in dry THF at 0 °C. After 45–50 min (TLC monitored) the THF was removed under vacuum. The crude allyl vinyl ether was extracted with ethyl acetate (3 × 100 ml). The combined organic layer was dried over anhydrous Na_2SO_4 and concentrated under vacuum. The crude product was purified by column chromatography with ethyl acetate–hexane as eluent to give allyl vinyl ether **5** as an inseparable mixture of geometrical isomers (5.4 g, 80%).

^{1}H NMR (300 MHz, $CDCl_3$) δ: 4.45 (d, *J* = 5.2 Hz, 1.34H, -C**H**$_2$CH=CH$_2$) *E*-isomer, 4.50 (d, *J* = 5.2 Hz, 0.66H, -C**H**$_2$CH=CH$_2$) *Z*-isomer, 5.34-5.50 (m, 2H, CH=C**H**$_2$), 5.75 (d, *J* = 7.3 Hz, 0.33H, -C**H**=CH-O-), 5.97–6.12 (m, 1H, C**H**=CH$_2$), 6.42 (d, *J* = 7.3 Hz, 0.33H, -CH=C**H**-O-), 6.50 (d, *J* =12.9 Hz, 0.67H, -C**H**=CH-O-), 7.08 (d, *J* =12.9 Hz, 0.67H, -CH=C**H**-O-), 7.27–7.35 (m, 1H, Ar-**H**), 7.49–7.58 (m, 2H, Ar-**H**), 7.92 (d, *J* = 8.0 Hz, 0.67H, Ar-**H**), 8.19 (d, *J* = 8.0 Hz, 0.33H, Ar-**H**). ^{13}C NMR (75 MHz, $CDCl_3$) δ: 150.93, 148.93, 147.14, 132.71, 132.43, 132.02, 131.72, 130.91, 129.76, 126.91, 126.24, 125.91, 124.83, 124.12, 118.30, 118.21, 101.86, 99.12, 74.22, 70.80. IR (neat): 2922, 2856, 1681, 1653, 1525, 1352, 916, 732 cm.$^{-1}$ GC–MS *m/z*: 205. Anal. Calcd for $C_{11}H_{11}NO_3$: C, 64.38; H, 5.40; N, 6.83. found: C, 64.46; H, 5.35; N, 6.82.

2-(2-Nitrophenyl)-pent-4-enal (6). Isomer mixture **5** (3 g, 0.014 mol) was refluxed in xylene (20 ml) for 6–7 h. Complete consumption of the starting material was observed by TLC. At this stage the reaction mixture was cooled to room temperature and concentrated under reduced pressure. The crude product was purified by silica gel column chromatography with hexane–ethyl acetate (98:2) as eluent to afford **6** (2.55 g, 85%) as a thick yellow liquid.

^{1}H NMR (300 MHz, $CDCl_3$) δ: 2.50–2.60 (m, 1H, -C**H**$_2$CH=CH$_2$), 2.90-2.99 (m, 1H, -C**H**$_2$CH=CH$_2$), 4.28–4.33 (m, 1H, Ar-C**H**), 4.99–5.05 (m, 2H, CH=C**H**$_2$), 5.63–5.77 (m, 1H, C**H**=CH$_2$), 7.28 (d, *J* = 8.2 Hz, 1H, Ar-**H**), 7.45 (t, *J* = 8.0 Hz, 1H, Ar-**H**), 7.61 (t, *J* = 7.4 Hz, 1H, Ar-**H**), 7.97 (d, *J* = 8.0 Hz, 1H, Ar-**H**), 9.78 (s, 1H, C**H**O). ^{13}C NMR (75 MHz, $CDCl_3$) δ: 198.29, 149.69, 133.85, 133.13 131.02, 130.85, 128.34, 124.93, 117.89, 53.41, 33.69. IR (neat): 2928, 2858, 2719, 1726, 1641, 1527, 1357, 921,

732 cm^{-1}.GC–MS *m/z*: 205. Anal. Calcd for $C_{11}H_{11}NO_3$: C, 64.38; H, 5.40; N, 6.83. found: C, 64.50; H, 5.32; N, 6.82.

2-(2-Nitrophenyl)-pent-4-enoic acid (7). Jones reagent (10 ml, 0.018 mol) was added dropwise to a solution of aldehyde **6** (2 g, 0.009 mol) in acetone (25 ml) at 0 °C and the resulting mixture stirred at room temperature for 5 h. The acetone was decanted and the green salt washed with acetone (2 ×10 ml). After evaporation of the combined organic extracts, water (25 ml) was added and the crude product extracted with (3 × 50 ml) ethyl acetate. The combined organic extracts were dried over anhydrous Na_2SO_4 and concentrated under reduced pressure. The crude compound was purified by silica-gel chromatography with ethyl acetate–hexane (5:95) as eluent to afford acid **7** (1.72 g, 80%).

^{1}H NMR (300 MHz, $CDCl_3$) δ: 2.65 (m, 1H, –C**H**$_2$CH=CH$_2$), 2.90 (m, 1H, –C**H**$_2$CH=CH$_2$), 4.32 (t, *J* = 7.0 Hz, 1H, Ar-C**H**), 4.98–5.06 (m, 2H, CH=C**H**$_2$), 5.70–5.78 (m, 1H, C**H**=CH$_2$), 7.43 (t, *J* = 8.2 Hz, 1H, Ar-**H**), 7.52 (d, *J* = 7.6 Hz, 1H, Ar-**H**), 7.59 (t, *J* = 7.6 Hz, 1H, Ar-**H**), 7.90 (d, *J* = 8.2 Hz, 1H, Ar-**H**), 9.17 (bs, 1H, COO**H**). ^{13}C NMR (75 MHz, $CDCl_3$) δ: 177.53, 149.13, 134.13, 133.10, 132.34, 130.42, 128.31, 124.80, 117.92, 46.38, 36.21. IR (neat): 3325, 2980, 2928, 1710, 1527, 922 cm^{-1}. GC–MS *m/z*: 221. Anal. Calcd for $C_{11}H_{11}NO_4$: C, 59.73; H, 5.01; N, 6.33. found: C, 59.81; H, 4.97; N, 6.31.

Ethyl 2-(2-nitrophenyl)-pent-4-enoate (8). To a solution of acid **7** (1.5 g, 0.006 mol) in dry ethanol (20 ml), was added a catalytic amount of concentrated sulfuric acid and the

resulting mixture heated under reflux for 1 h. The ethanol was then removed under reduced pressure. The residue was extracted with (3 × 50 ml) ethyl acetate, washed with brine, dried over anhydrous Na_2SO_4 and concentrated under reduced pressure. The crude product was purified by chromatography on silica with ethyl acetate–hexane (3:97) as eluent to furnish ester **8** (1.38 g, 82%).

^{1}H NMR (300 MHz, $CDCl_3$) δ: 1.19 (t, *J* = 7.1 Hz, 3H, -$CH_2C\mathbf{H}_3$), 2.57–2.67 (m, 1H, -$C\mathbf{H}_2CH=CH_2$), 2.87–2.96 (m, 1H, -$C\mathbf{H}_2CH=CH_2$), 4.09–4.17 (q, *J* = 4.4 Hz, 2H, -$C\mathbf{H}_2CH_3$), 4.29 (t, *J* = 7.7 Hz, 1H, Ar-C**H**), 4.98–5.08 (m, 2H, $CH=C\mathbf{H}_2$), 5.70–5.79 (m, 1H, $C\mathbf{H}=CH_2$), 7.39–7.44 (t, *J* = 7.2 Hz, 1H, Ar-**H**), 7.52–7.59 (m, 2H, Ar-**H**), 7.87–7.90 (d, *J* = 8.2 Hz, 1H, Ar-**H**). ^{13}C NMR (75 MHz, $CDCl_3$) δ: 171.92, 149.23, 134.45, 132.93, 132.83, 129.97, 127.97, 124.60, 117.56, 61.11, 46.10, 36.70, 13.91. IR (neat): 2982, 1735, 1529, 1352, 916, 736 cm^{-1}. GC–MS *m/z*: 249. Anal. Calcd for $C_{13}H_{15}NO_4$: C, 62.64; H, 6.07; N, 5.62. found: C, 62.72; H, 6.01; N, 5.60.

3-Allylindolin-2-one (9). To a mixture of compound **8** (1.4 g, 0.0056 mol) in ethanol (8 ml) and water (2 ml), ammonium chloride (1.19 g, 0.022 mol) and zinc powder (1.09 g, 0.016 mol) were added and the mixture was stirred vigorously at reflux for 30 min. When the reaction was complete (TLC), the mixture was filtered, and the ethanol removed under reduced pressure. The residue was extracted with ethyl acetate (3 × 20 ml), washed with brine, dried over anhydrous Na_2SO_4. After removal of the solvent under reduced pressure, the residue was purified by column chromatography with ethyl acetate–hexane (4:96) as eluent to give the oxindole **9** (0.75 g, 78%).

^{1}H NMR (300 MHz, $CDCl_3$) δ: 2.53–2.63 (m, 1H, –C**H**$_2$CH=CH$_2$), 2.79–2.87 (m, 1H, -C**H**$_2$CH=CH$_2$), 3.50–3.54 (t, *J* = 6.6 Hz, 1H, Ar-C**H**), 5.02–5.14 (m, 2H, CH=**CH$_2$**), 5.69–5.83 (m, 1H, C**H**=CH$_2$), 6.91–7.01 (m, 2H, Ar-**H**), 7.16–7.25 (m, 2H, Ar-**H**), 9.85 (bs, 1H, N**H**). ^{13}C NMR (75 MHz, $CDCl_3$) δ: 180.53, 141.70, 133.74, 129.12, 127.81, 124.17, 121.99, 117.93, 109.86, 45.71, 34.58. IR (neat): 3280, 2922, 1708, 1620, 1469, 750 cm^{-1}. GC–MS *m/z*: 173. Anal. Calcd for $C_{11}H_{11}NO$: C, 76.28; H, 6.40; N, 8.09. found: C, 76.37; H, 6.34; N, 8.04.

***tert*-Butyl 3-allyl-2-oxoindoline-1-carboxylate (10)**. Boc anhydride (0.943 g, 4.33 mmol) was added dropwise at 0 °C to an ice cold suspension of sodium hydride (104 mg, 4.33 mmol) in a solution of **9** (750 mg, 4.33 mmol) in THF (10 ml). The resulting mixture was stirred for 5 min at 0 °C then poured into cold water. The aqueous layer was extracted with ethyl acetate (3 × 25 ml). The combined organic layers were dried over Na_2SO_4, filtered, and concentrated in vacuo. The crude material was purified by column chromatography with acetone–hexane (1:99) as eluent to give **10** (826 mg, 70%) as a thick pale yellow oil.

^{1}H NMR (300 MHz, $CDCl_3$) δ: 1.64 (s, 9H, OCC**H**$_3$), 2.58–2.68 (m, 1H, –C**H**$_2$CH=CH$_2$), 2.79–2.88 (m, 1H, –C**H**$_2$CH=CH$_2$), 3.59–3.63 (t, *J* = 6.5 Hz, 1H, Ar-C**H**), 5.05–5.14 (m, 2H, –CH=C**H**$_2$), 5.68–5.79 (m, 1H, –C**H**=CH$_2$), 7.11–7.16 (t, *J* = 7.6 Hz, 1H, Ar-**H**), 7.27–7.32 (m, 2H, Ar-**H**), 7.79–7.82 (d, *J* = 8.2 Hz, 1H, Ar-**H**). ^{13}C NMR (75 MHz, $CDCl_3$) δ: 175.29, 149.02, 139.88, 133.27, 127.96, 127.20, 123.96, 123.82, 118.36, 114.70, 84.03, 45.48, 35.25, 27.89. IR (neat): 2926, 1767, 1732, 1462, 1151., 736 cm^{-1}. GC–MS *m/z*:

273. Anal. Calcd for $C_{16}H_{19}NO_3$: C, 70.31; H, 7.01; N, 5.12. found: C, 70.39; H, 6.97; N, 5.09.

1-*tert*-Butyl 3-ethyl 3-allyl-2-oxoindoline-1,3-dicarboxylate (11). Ethyl chloroformate (316 mg, 2.93 mmol) was added dropwise at 0 °C to an ice cold suspension of sodium hydride (70 mg, 2.93 mmol) in a solution of **10** (800 mg, 2.93 mmol) in THF (10 ml). The resulting mixture was stirred at room temperature. After 2 h (TLC) the THF was removed under vacuum. The crude reaction mixture was extracted with ethyl acetate (3 × 25 ml). The combined organic layers were washed with brine, dried over anhydrous Na_2SO_4 and concentrated under vacuum. The residue was purified by column chromatography with ethyl acetate–hexane (2:98) as eluent to afford compound **11** (808 mg, 80%) as a thick yellow liquid.

^{1}H NMR (300 MHz, $CDCl_3$) δ: 1.37–1.42 (t, *J* = 7.0 Hz, 3H, –$OCH_2C\mathbf{H}_3$), 1.63 (s, 9H, $OCC\mathbf{H}_3$), 3.36–3.38 (d, *J* = 5.9 Hz, 2H, –$C\mathbf{H}_2CH{=}CH_2$), 4.31–4.38 (q, *J* = 7.6 Hz, 2H, -$OC\mathbf{H}_2CH_3$), 5.03–5.16 (m, 2H, $CH{=}C\mathbf{H}_2$), 5.86–5.99 (m, 1H, $C\mathbf{H}{=}CH_2$), 7.20–7.34 (m, 2H, Ar-H), 7.46–7.49 (d, *J* = 7.0 Hz, 1H, Ar-H), 8.06–8.08 (d, *J* = 8.2 Hz, 1H, Ar-**H**). ^{13}C NMR (75 MHz, $CDCl_3$) δ: 152.27, 148.78, 137.91, 134.75, 132.20, 126.91, 124.22, 122.77, 118.98, 115.95, 115.31, 106.10, 84.17, 65.53, 28.03, 26.78, 14.04. IR (neat): 2926, 1798, 1775, 1735, 1462, 1151., 736 cm^{-1}. GC–MS *m/z*: 345. Anal. Calcd for $C_{19}H_{23}NO_5$: C, 66.07; H, 6.71; N, 4.06. found: C, 66.19; H, 6.65; N, 4.01.

1-*tert*-Butyl 3-ethyl 2-oxo-3-(2-oxoethyl)indoline-1,3-dicarboxylate (12). To a stirred solution of **11** (800 mg, 2.31 mmol) in aqueous THF (H_2O:THF (2 ml:8 ml)) at room

temperature, was added a catalytic amount of potassium osmate (1 mol%) and *N*-methylmorpholine-*N*-oxide (542 mg, 4.62 mmol). The solution was stirred for 6 h at room temperature. After 6 h (TLC) the THF was removed under vacuum. The crude reaction mixture was extracted with ethyl acetate (3 × 25 ml). The combined organic layers were dried over anhydrous Na_2SO_4 and concentrated under vacuum to give the crude diol. Sodium periodate on silica [#] (494 mg, 2.31 mmol) was added to a stirred solution of the diol in DCM at room temperature. The reaction was stirred for 15 min and then filtered through a silica gel plug with which as washed with ethyl acetate. The ethyl acetate was removed in vacuo to give crude **12**. The crude product was purified by column chromatography with ethyl acetate–hexane (5:95) as eluent to afford compound **12** (643 mg, 80%) as a thick yellow liquid.

[#]. Preparation of silica gel supported $NaIO_4$:- Sodium meta periodate (1 g, 4.69 mmol) was dissolved in hot water (2 ml). To the hot solution, silica gel (230–400 mesh, 4 g) was added with vigorous swirling and shaking. The resultant silica gel coated with $NaIO_4$ was obtained as a free flowing powder.

^{1}H NMR (300 MHz, $CDCl_3$) δ: 1.38–1.43 (t, *J* =7.3 Hz, 3H, $OCH_2C\mathbf{H}_3$), 1.65 (s, 9H, $OCC\mathbf{H}_3$), 3.64–3.65 (d, *J* = 2.4 Hz, 2H, $C\mathbf{H}_2CHO$), 4.33–4.40 (q, *J* = 7.3 Hz, 2H, $OC\mathbf{H}_2CH_3$), 7.26–7.41 (m, 3H, Ar-**H**), 8.07–8.10 (d, *J* = 7.3 Hz, 1H, Ar-**H**), 9.66 (s, 1H, C**H**O). ^{13}C NMR (75 MHz, $CDCl_3$) δ: 197.80, 152.15, 148.52, 139.47, 132.14, 126.39, 124.78, 123.29, 118.40, 115.52, 99.55, 84.79, 65.96, 37.51, 28.03, 14.00. IR (neat): 2982, 1778, 1775, 1735, 1458, 1246., 756 cm^{-1}. GC–MS *m/z*: 347. Anal. Calcd for $C_{18}H_{21}NO_6$: C, 62.24; H, 6.09; N, 4.03. found: C, 62.35; H, 6.00; N, 4.01.

***tert*-Butyl 1'-methyl-2,2'-dioxospiro[indoline-3,3'-pyrrolidine]-1-carboxylate (13)**. A solution of aldehyde **12** (500 mg, 1.44 mmol) in THF (20 ml) was cooled to 0 °C and

magnesium sulfate (1.72 g, 14.4 mmol) followed by methylamine hydrochloride (386 mg, 5.76 mmol) and Et_3N (0.6 ml, 5.76 mmol) was added. The reaction mixture was stirred for 1.5 h at room temperature. Sodium cyanoborohydride (2.88 mmol) was added at 0 °C and the mixture stirred at room temperature for 2 h. The reaction was quenched with 1 M NaOH and extracted with DCM (3 × 10ml). The combined DCM layers were dried over anhydrous Na_2SO_4, filtered and concentrated in vacuo. The crude material was purified by silica gel chromatography to afford **13** (273 mg, 60%) as a thick pale yellow oil.

^{1}H NMR (300 MHz, $CDCl_3$) δ: 1.53 (s, 9H, –OCC**H**$_3$), 2.37 (m, 1H, C-4'-**H**), 2.95 (s, 3H, NC**H**$_3$), 3.13 (m, 1H, C-4'-**H**), 3.54 (m, 1H, C-3'-**H**), 4.00 (m, 1H, C-3'-**H**), 7.09–7.14 (t, *J* = 7.3 Hz, 1H, Ar-**H**), 7.22–7.25 (d, *J* = 8.6 Hz, 1H, Ar-**H**), 7.28–7.33 (t, *J* = 7.3 Hz, 1H, Ar-**H**), 7.75–7.78 (d, *J* = 8.2 Hz, 1H, Ar-**H**). ^{13}C NMR (75 MHz, $CDCl_3$) δ: 174.23, 159.78, 147.55, 136.71, 127.20, 124.95, 123.50, 118.40, 115.80, 95.21, 84.50, 47.05, 30.85, 29.15, 28.05. IR (neat): 2980, 1732, 1460, 1240, 760 cm^{-1}. GC–MS *m/z*: 316. Anal. Calcd for $C_{17}H_{20}N_2O_4$: C, 64.54; H, 6.37; N, 8.86. found: C, 64.62; H, 6.31; N, 8.83.

1'-Methylspiro[indoline-3,3'-pyrrolidine]-2,2'-dione (14). To a stirred solution of **13** (270 mg, 0.854 mmol) in THF (5 ml), was added 2.5 M HCl (2 ml) and the mixture heated under reflux for 30 min. The reaction was then quenched by the addition of saturated $NaHCO_3$ and extracted with EtOAc (3 × 25 ml). The combined organic layers were dried over anhydrous Na_2SO_4 and conctentrated under vacuum. The crude

material was purified by chromatography with methanol–chloroform (5:95) as eluent to afford **14** (158 mg, 86%) as a viscous yellow oil.

^{1}H NMR (300 MHz, $CDCl_3$) δ: 2.32 (m, 1H, C-4'-**H**), 2.91 (s, 3H, NC**H**$_3$), 3.01 (m, 1H, C-4'-**H**), 3.51 (m, 1H, C-3'-**H**), 3.91 (m, 1H, C-3'-**H**), 6.77 (m, 2H, Ar-**H**), 7.03 (t, *J* = 8.8 Hz, 1H, Ar-**H**), 7.28 (d, *J* = 8.2 Hz, 1H, Ar-**H**), 8.10 (bs, 1H, N**H**). ^{13}C NMR (75 MHz, $CDCl_3$) δ: 176.31, 170.54, 140.70, 130.93, 127.81, 124.17, 121.08, 118.10, 84.50, 48.36, 31.58, 30.11. IR (neat): 3215, 2982, 1710, 1469, 1240, 750 cm^{-1}. GC–MS *m/z*: 216. Anal. Calcd for $C_{12}H_{12}N_2O_2$: C, 66.65; H, 5.59; N, 12.96. found: C, 66.78; H, 5.51; N, 12.93.

Coerulescine (1). To an ice cold solution of **14** (150 mg, 0.694 mmol) in dry THF (15 ml), was added *n*-butyl lithium (1.6 ml, 0.694 mmol, 2.5 M solution in hexane) and the reaction mixture was stirred at 0 °C for 30 min. Then the LAH (52 mg, 1.38 mmol) was added at 0 °C and the mixture allowed to warm to room temperature then stirred for 1 h. The reaction was then quenched with water, filtered through Na_2SO_4 and concentrated in vacuo. The crude material was purified by silica gel chromatography with chloroform–methanol (5:95) as eluent to yield coerulescine (42 mg, 30%) as a pale yellow gum.

^{1}H NMR (300 MHz, $CDCl_3$) δ: 2.07 (m, 1H, C-4'-**H**), 2.40 (m, 1H, C-4'-**H**), 2.48 (s, 3H, NC**H**$_3$), 2.75–3.05 (m, 4H, C-1' & C-3'-**H**), 6.89 (d, *J* = 7.8 Hz, 1H, Ar-**H**), 7.04 (t, *J* = 7.3 Hz, 1H, Ar-**H**), 7.20 (t, *J* = 7.5 Hz, 1H, Ar-**H**), 7.41 (d, *J* = 7.3 Hz, 1H, Ar-**H**), 8.61 (bs, 1H, N**H**). ^{13}C NMR (75 MHz, $CDCl_3$) δ: 183.28, 140.38, 136.02, 127.81, 123.29, 122.84, 109.75, 66.12, 56.68, 53.65, 41.78, 37.88. IR (neat): 3212, 2978, 1709, 1465, 1238, 760

cm^{-1}. GC–MS *m/z*: 202. Anal. Calcd for $C_{12}H_{14}N_2O$: C, 71.26; H, 6.98; N, 13.85. found: C, 71.34; H, 6.91; N, 13.80.

Horsfiline (2). *N*-Bromosuccinimide (38 mg, 0.217 mmol) was added to a solution of **1** (40 mg, 0.198 mmol) in DMF (2 ml) at 0 °C. The mixture was stirred for 2 h at 0 °C. After the addition of water, the solution was extracted with ether. The ethereal extract was dried over Na_2SO_4 and concentrated. To a suspension of the crude product and CuI (41 mg, 0.217 mmol) in DMF (2 ml), was added a solution of sodium methoxide (0.2 ml, 0.297 mmol, 0.01 M). After stirring at 120 °C for 2 h, the reaction mixture was cooled and the insoluble materials removed by filtration. The filtrate was concentrated in vacuo and water added to the residue. The aqueous layer was extracted with ether, the extract washed with brine, dried over Na_2SO_4 and concentrated under reduced pressure. The residue was purified by column chromatography on silica gel with methanol- chloroform (5:95) as eluent to give horsfiline (27 mg, 60%) as light yellow crystals. mp 152–154 °C (lit 156–157 °C).

^{1}H NMR (300 MHz, $CDCl_3$) δ: 2.07–2.12 (m, 1H, C-4'-**H**), 2.37–2.45 (m, 1H, C-4'-**H**), 2.49 (s, 3H, NC**H**$_3$), 2.77–2.83 (m, 1H, C-3'-**H**), 2.88 (s, 2H, C-1'-**H**), 2.99–3.07 (m, 1H, C-3'-**H**), 3.80 (s, 3H, OC**H**$_3$), 6.72 (d, *J* = 8.0 Hz, 1H, Ar-**H**), 6.78 (d, *J* = 8.0 Hz, 1H, Ar-**H**), 7.05 (d, *J* = 2.0 Hz, 1H, Ar-**H**), 8.20 (bs, 1H, N**H**). ^{13}C NMR (75 MHz, $CDCl_3$) δ: 182.95, 156.28, 137.31, 133.47, 112.53, 110.27, 109.92, 66.30, 56.58, 56.02, 54.18, 41.78, 38.25. IR (neat): 3215, 2950, 1708, 1482, 760 cm^{-1}. GC–MS *m/z*: 232. Anal. Calcd for $C_{13}H_{16}N_2O_2$: C, 67.22; H, 6.94; N, 12.06. found: C, 67.36; H, 6.89; N, 12.00.

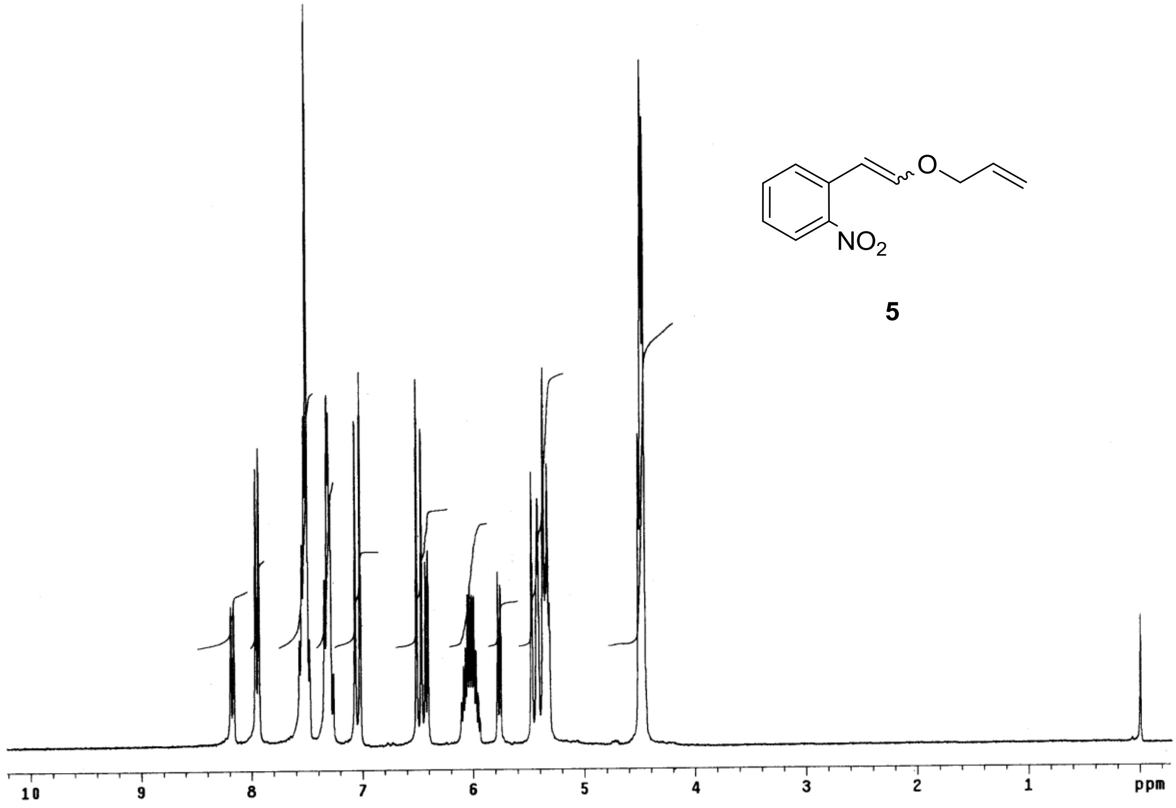

^{1}H NMR (300 MHz) Spectrum of the compound **5**

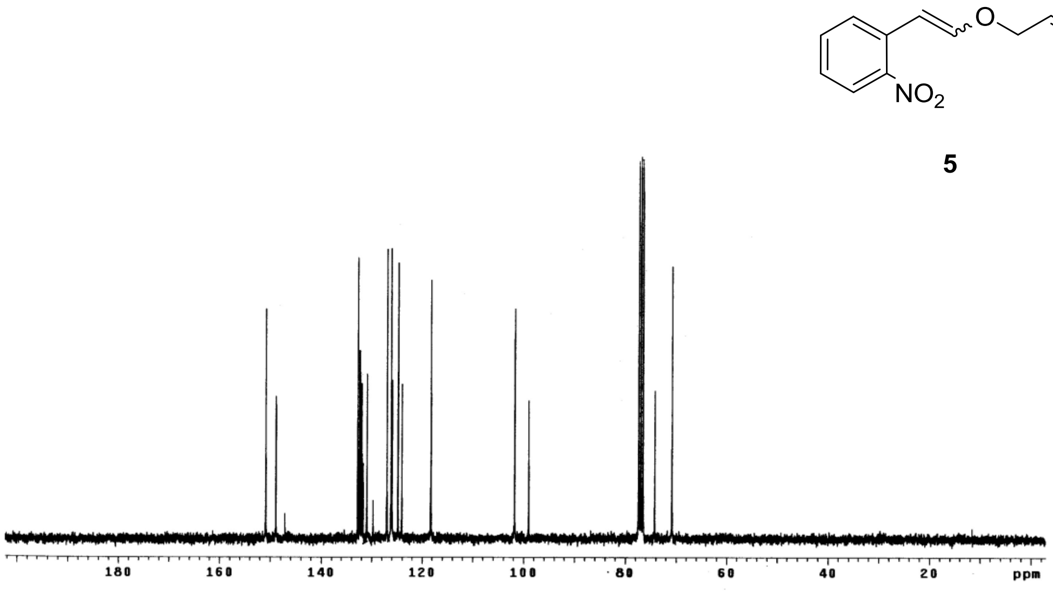

^{13}C NMR (75 MHz) Spectrum of the compound **5**

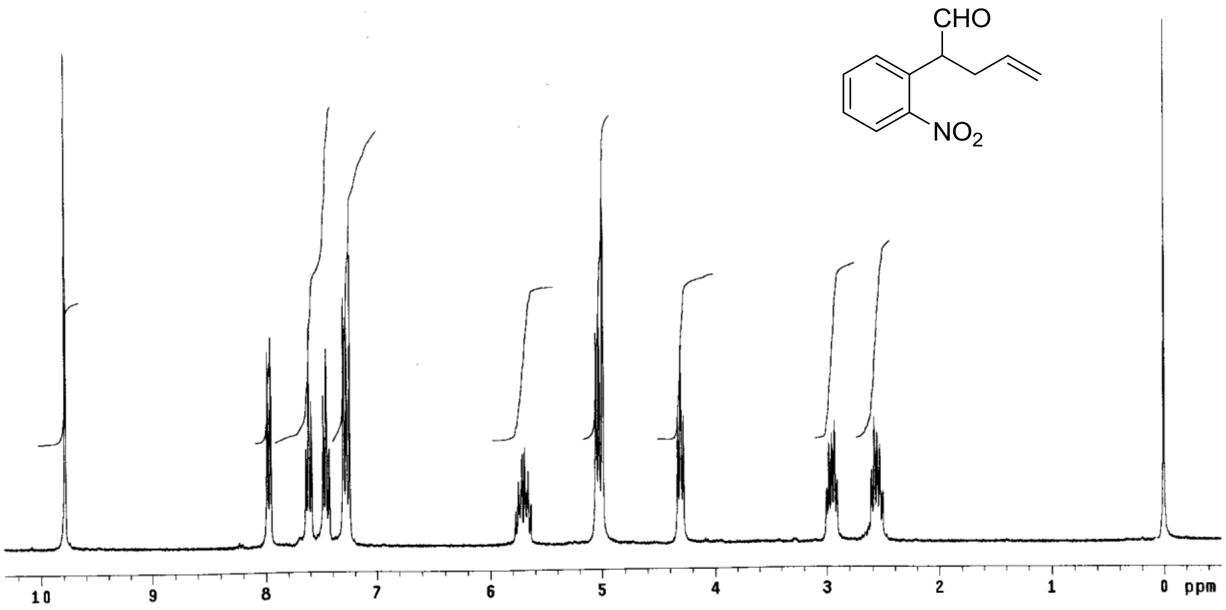

^{1}H NMR (300 MHz) Spectrum of the compound **6**

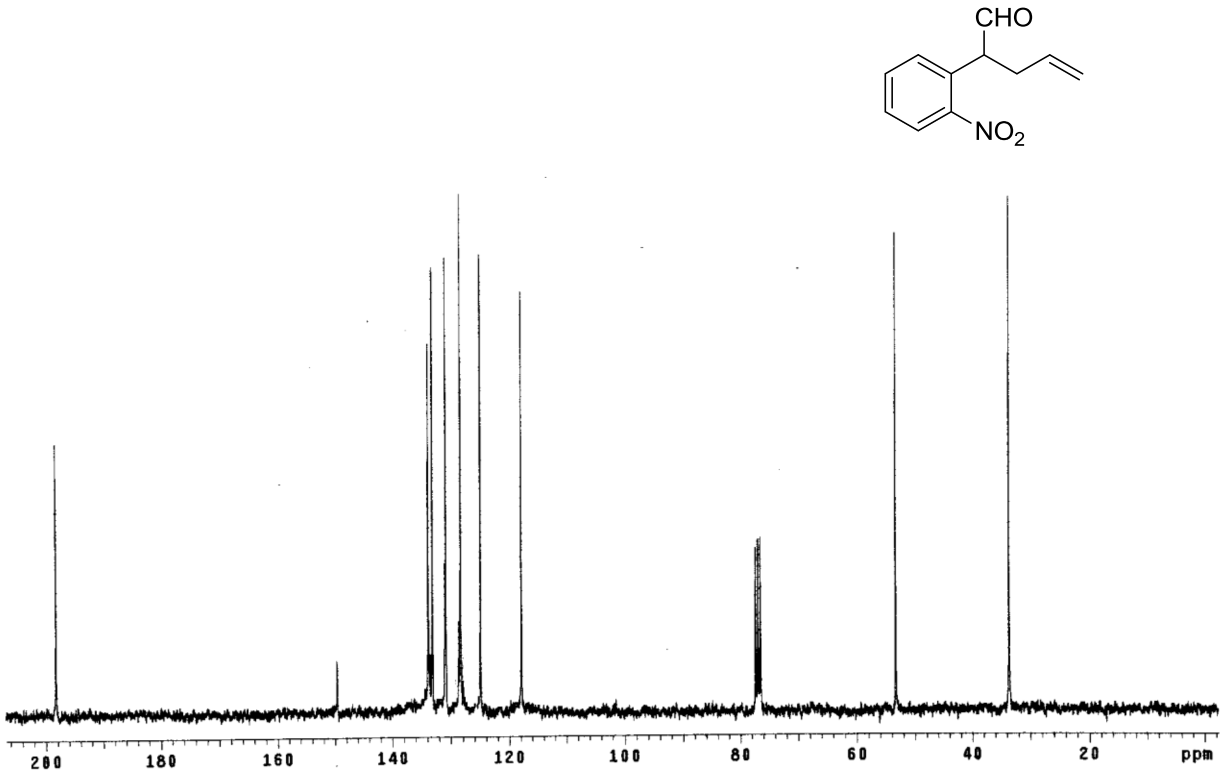

^{13}C NMR (75 MHz) Spectrum of the compound **6**

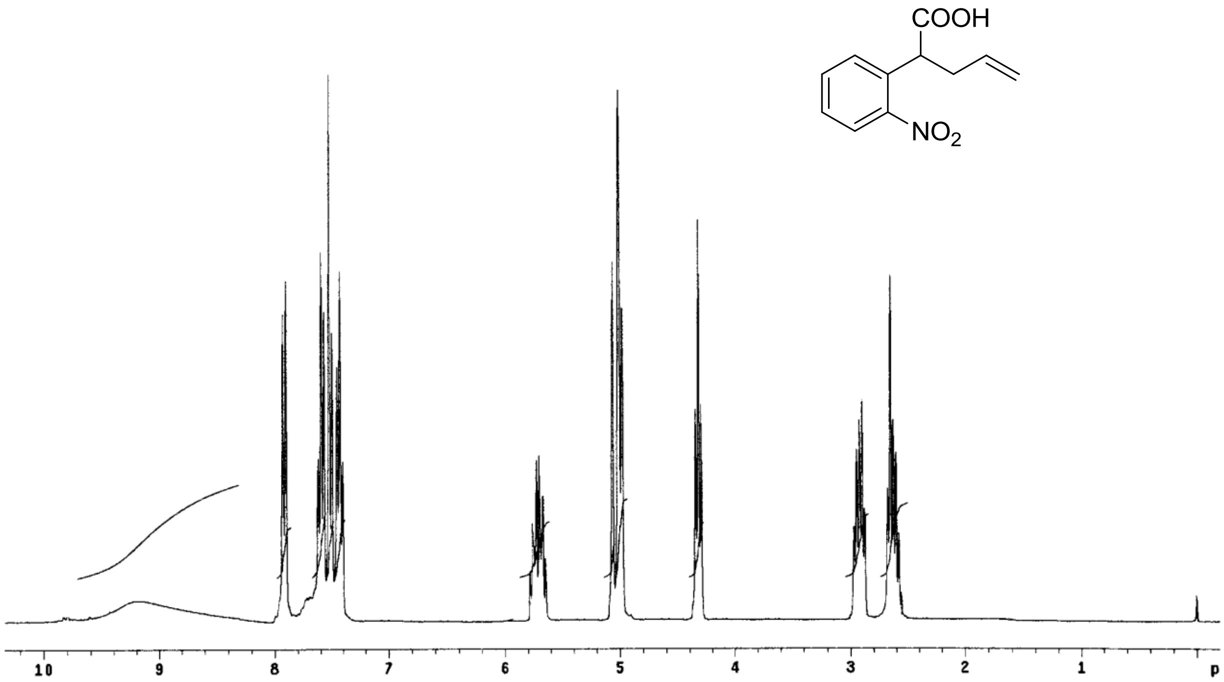

^{1}H NMR (300 MHz) Spectrum of the compound **7**

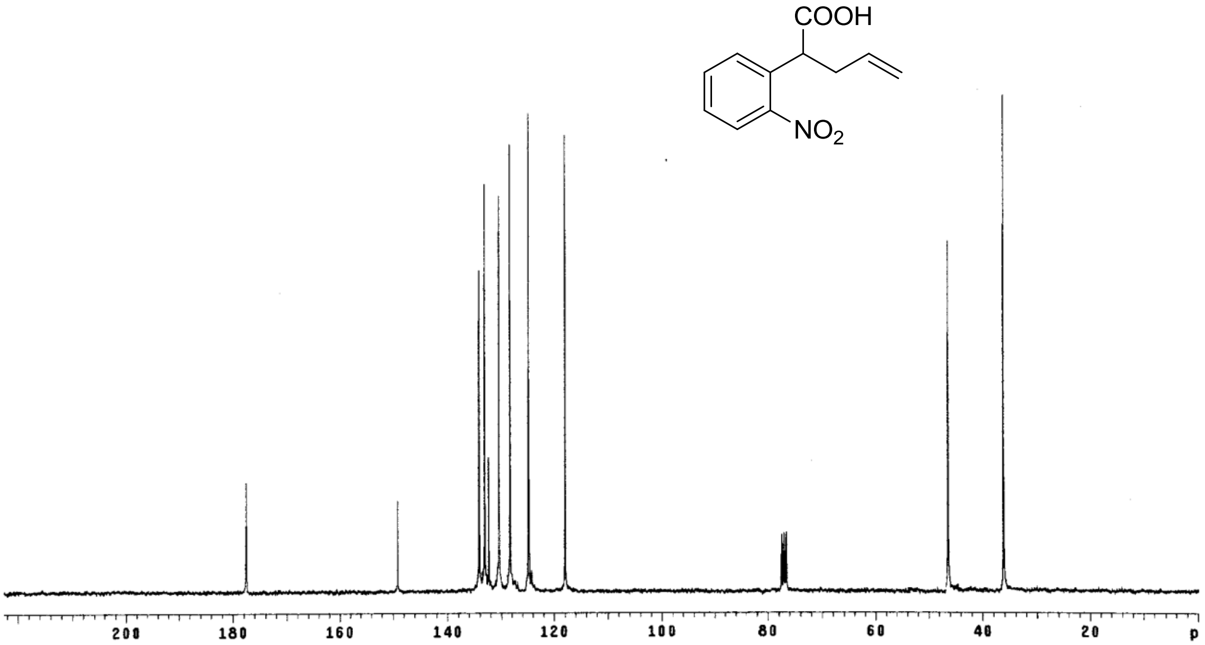

^{13}C NMR (75 MHz) Spectrum of the compound **7**

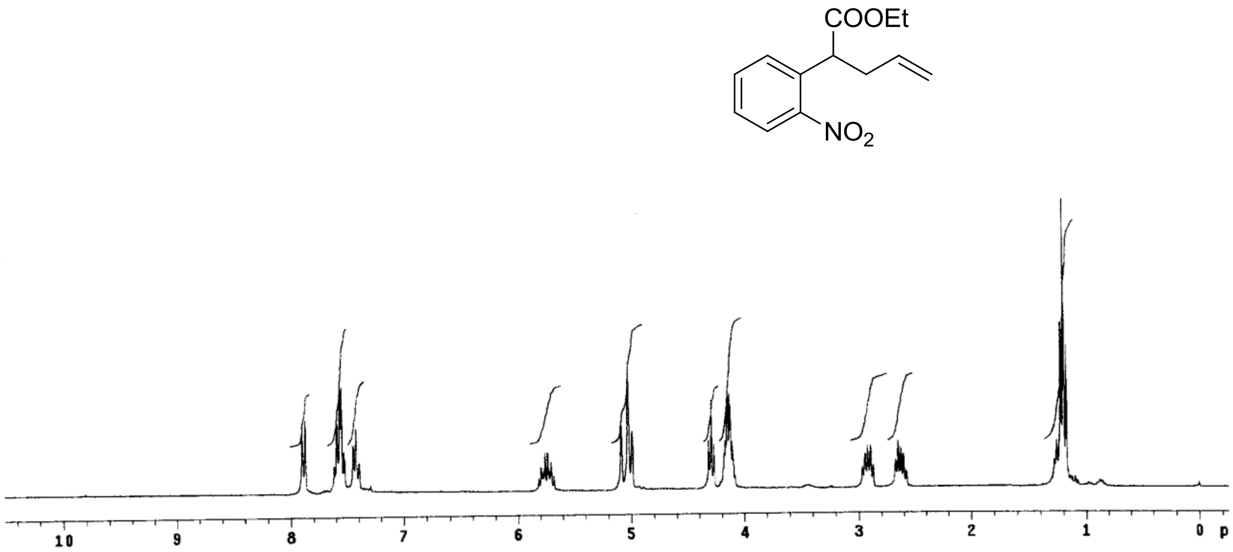

^{1}H NMR (300 MHz) Spectrum of the compound **8**

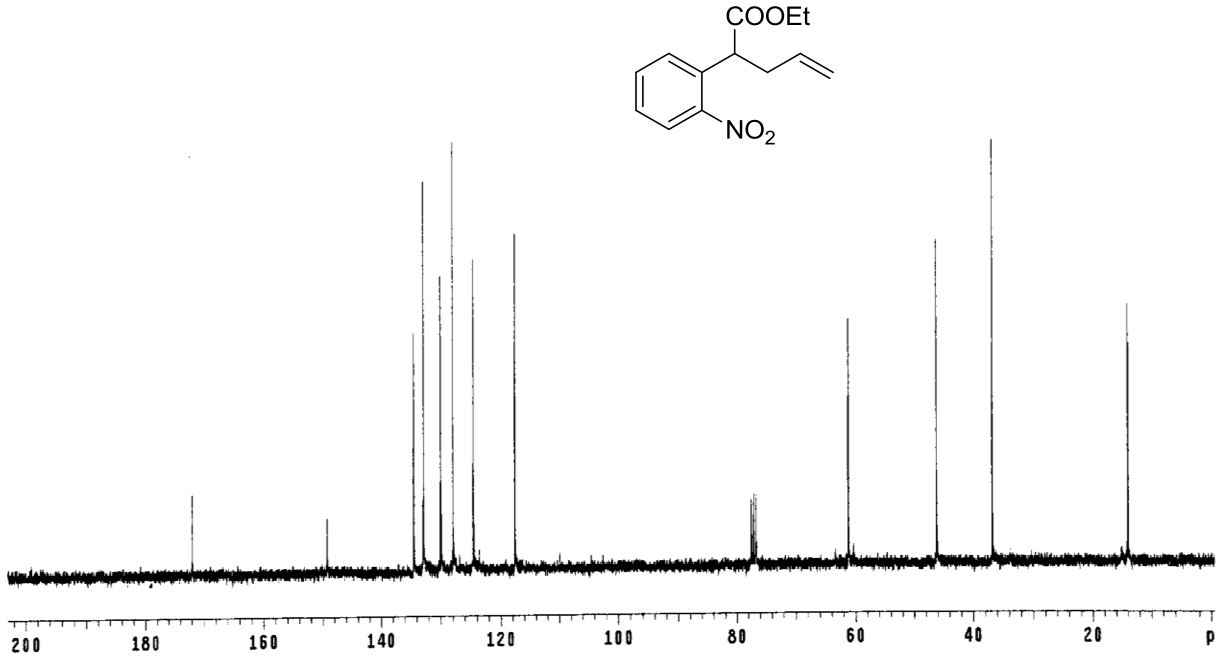

^{13}C NMR (75 MHz) Spectrum of the compound **8**

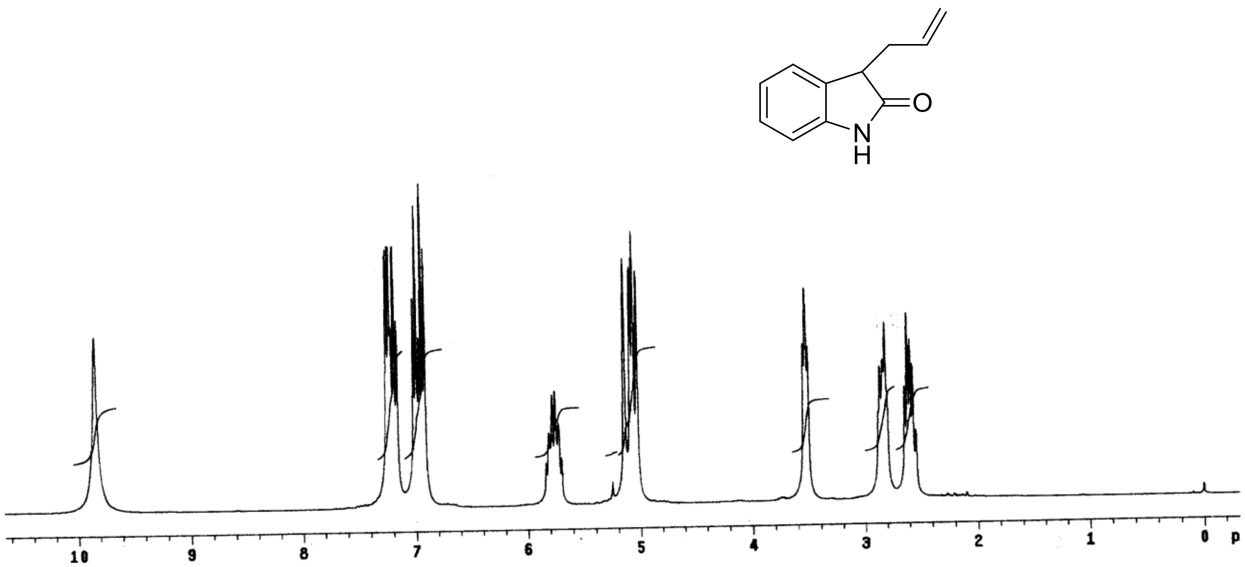

^{1}H NMR (300 MHz) Spectrum of the compound **9**

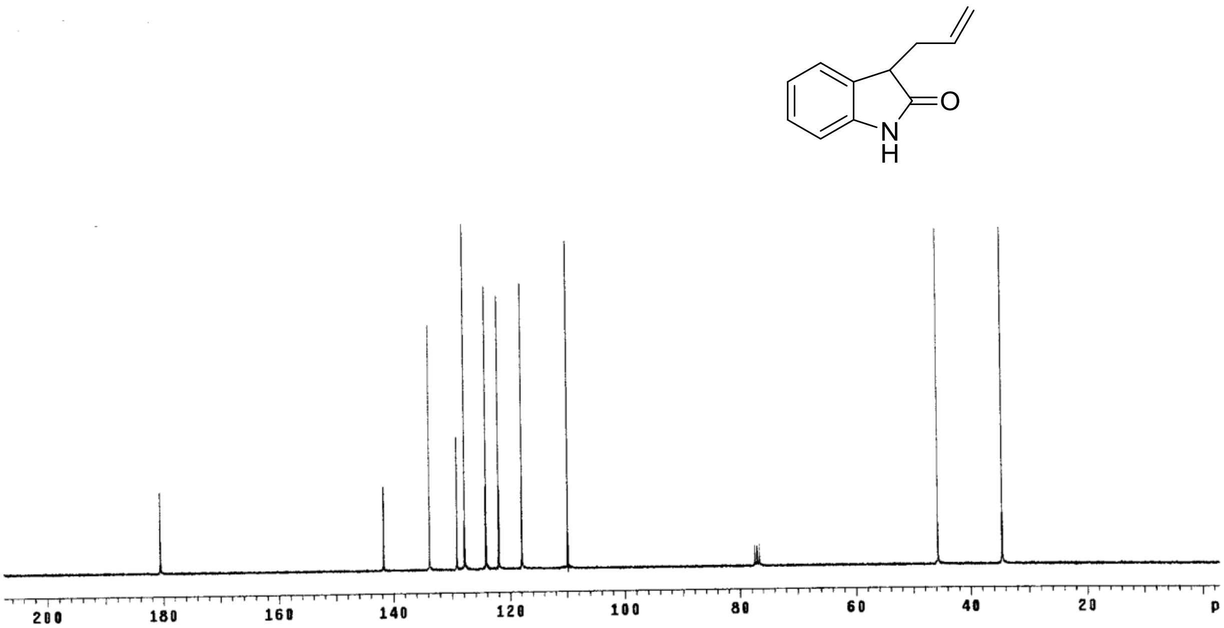

^{13}C NMR (75 MHz) Spectrum of the compound **9**

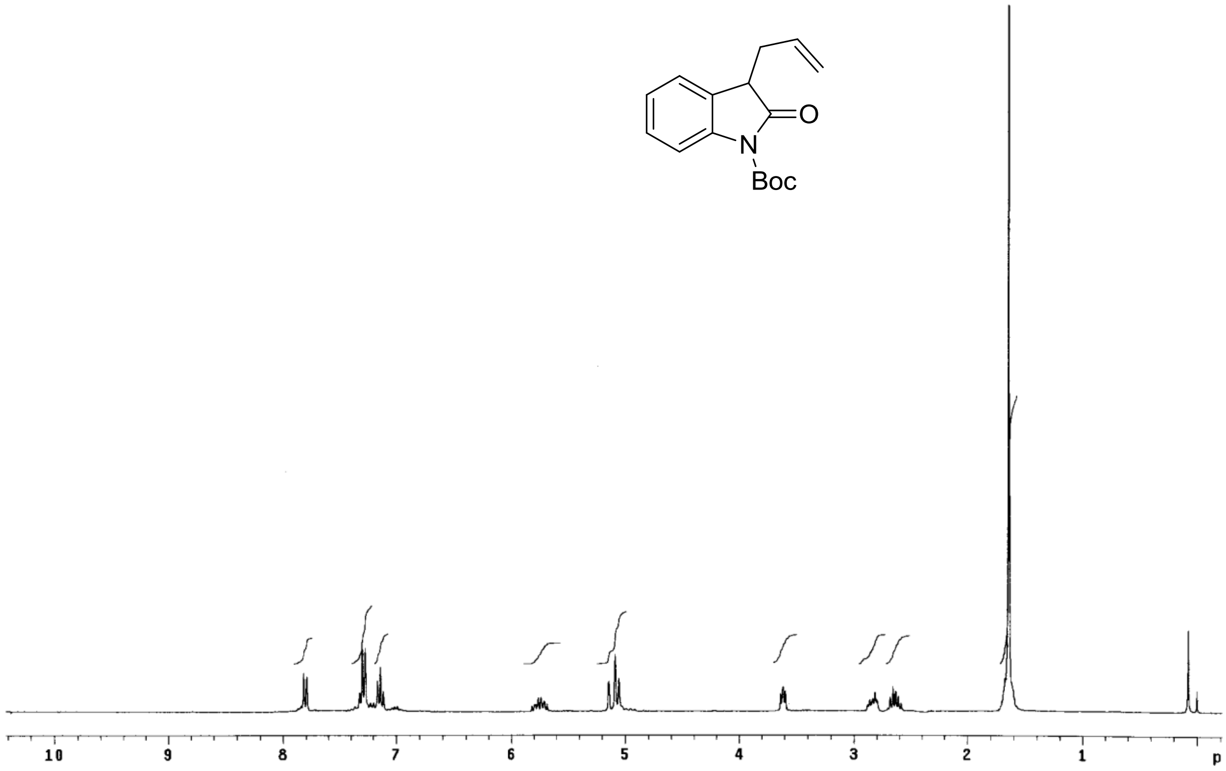

^{1}H NMR (300 MHz) Spectrum of the compound **10**

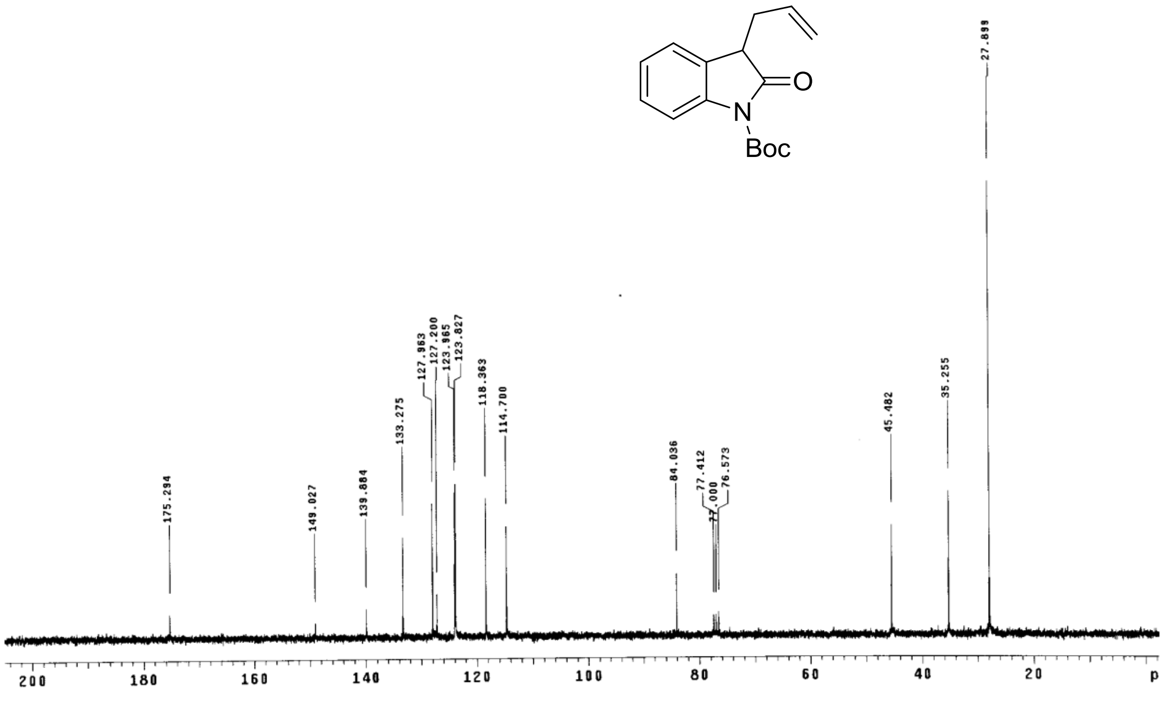

^{13}C NMR (75 MHz) Spectrum of the compound **10**

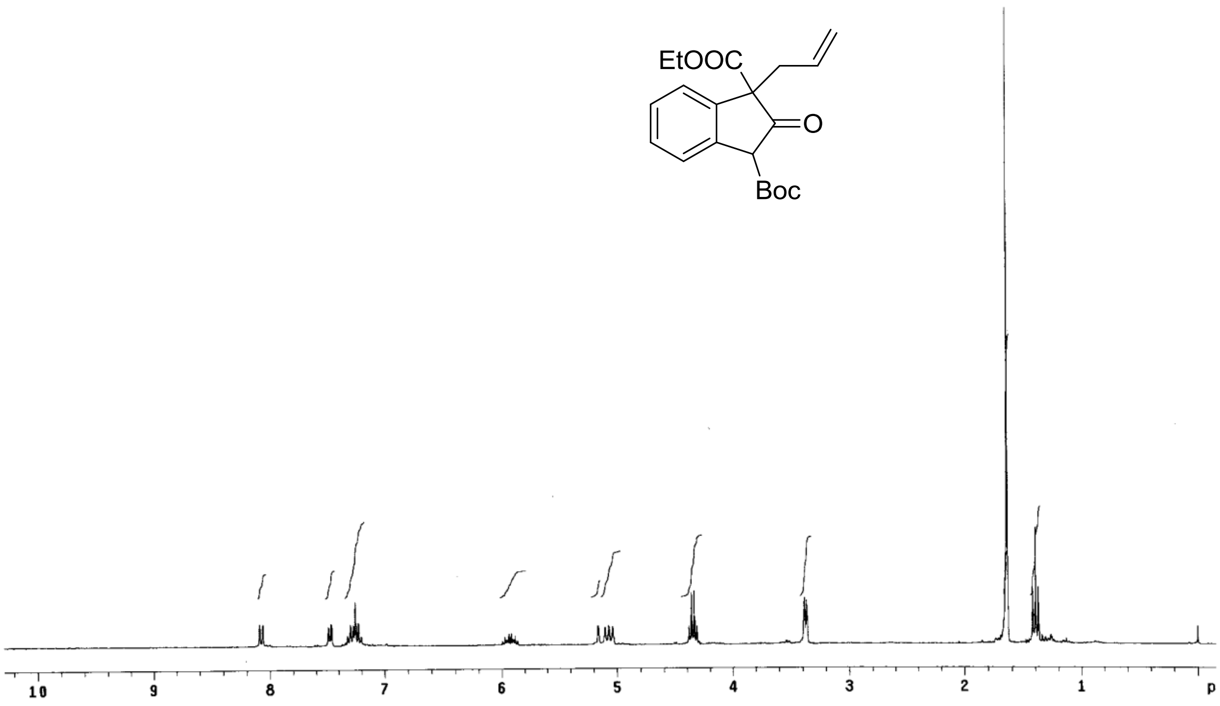

^{1}H NMR (300 MHz) Spectrum of the compound **11**

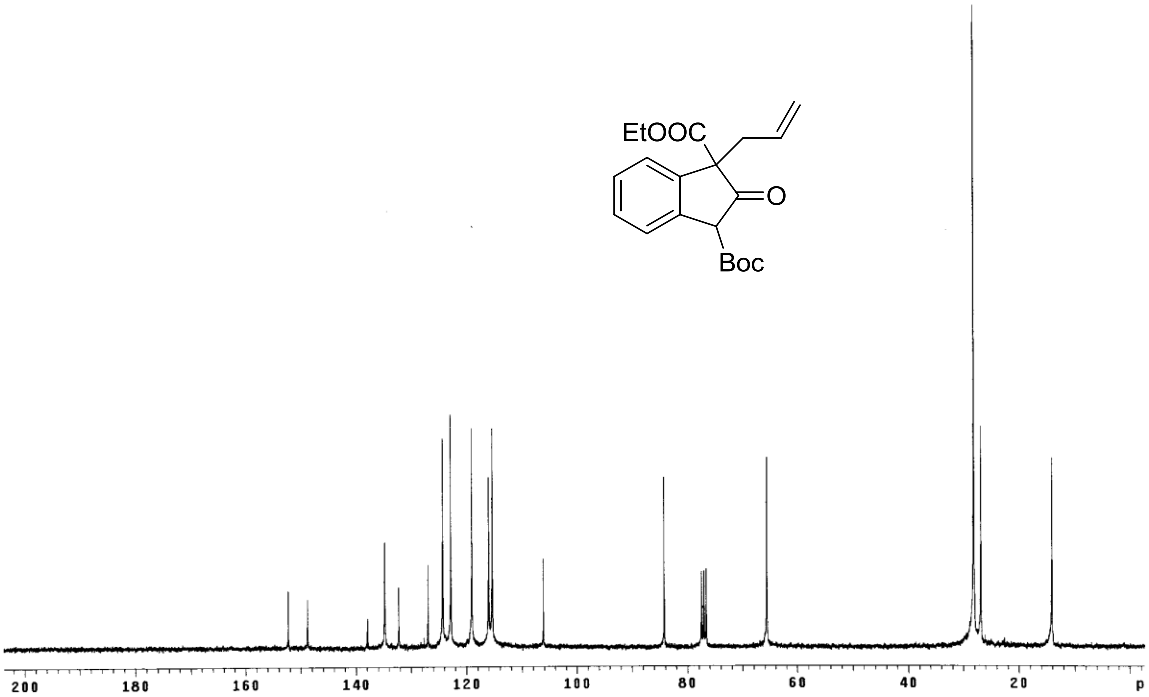

^{13}C NMR (75 MHz) Spectrum of the compound **11**

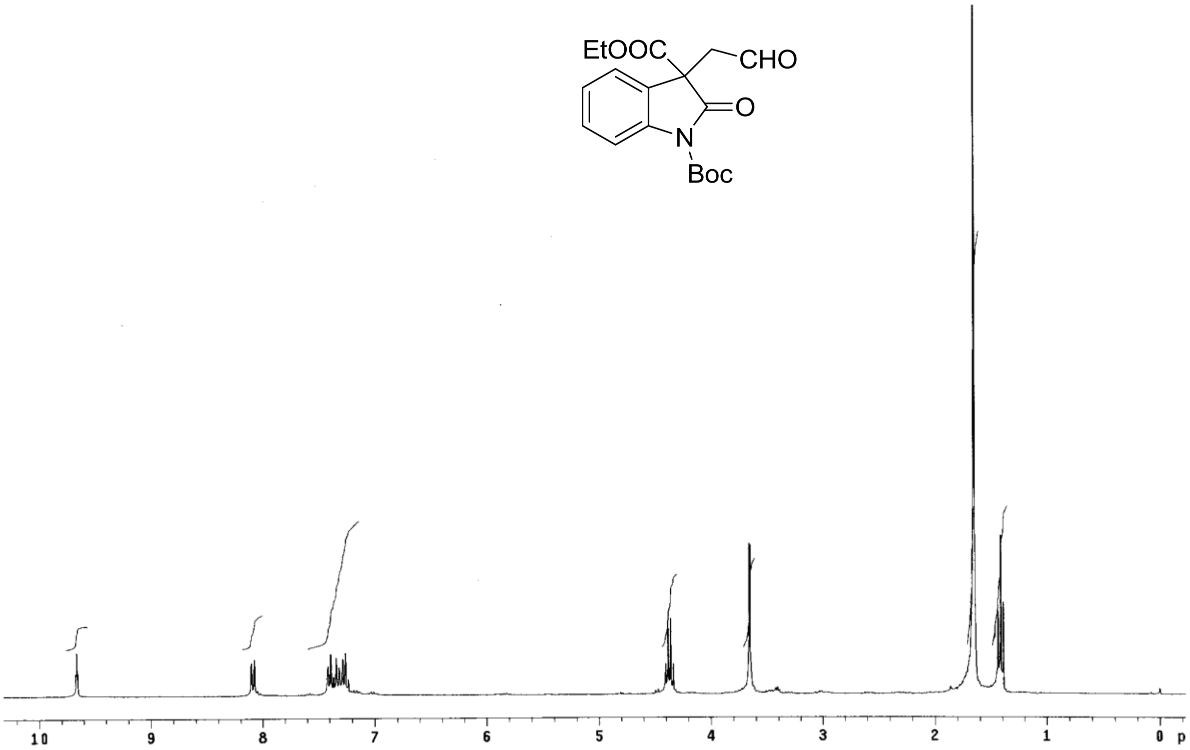

^{1}H NMR (300 MHz) Spectrum of the compound **12**

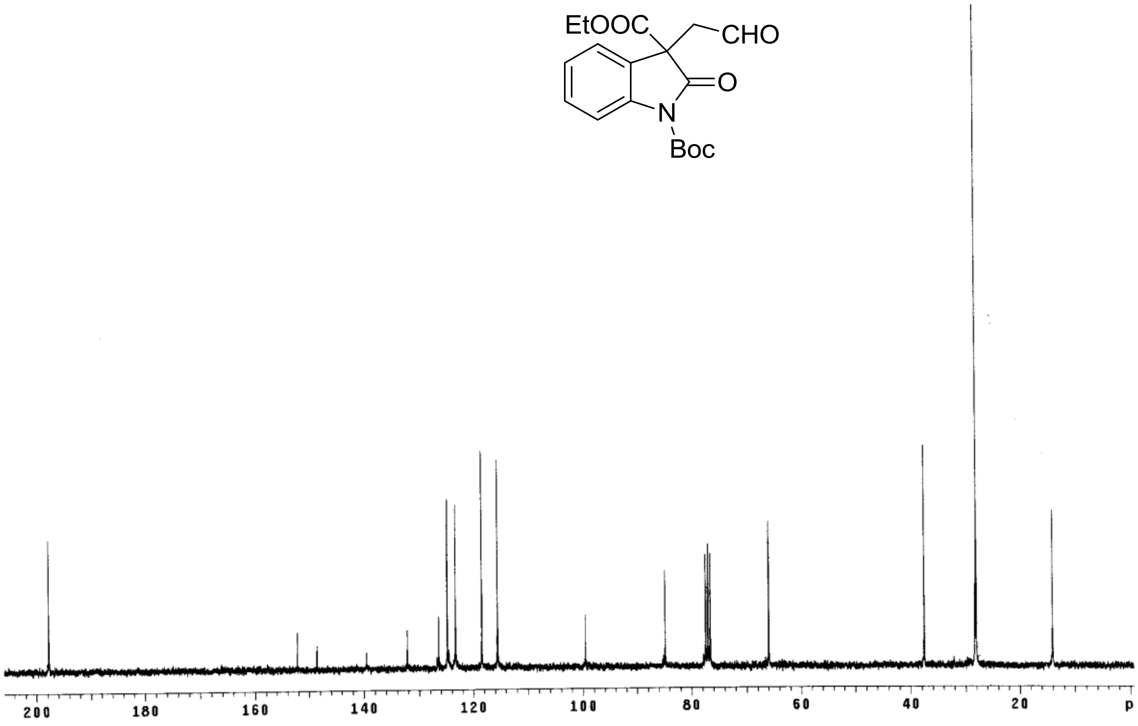

^{13}C NMR (75 MHz) Spectrum of the compound **12**

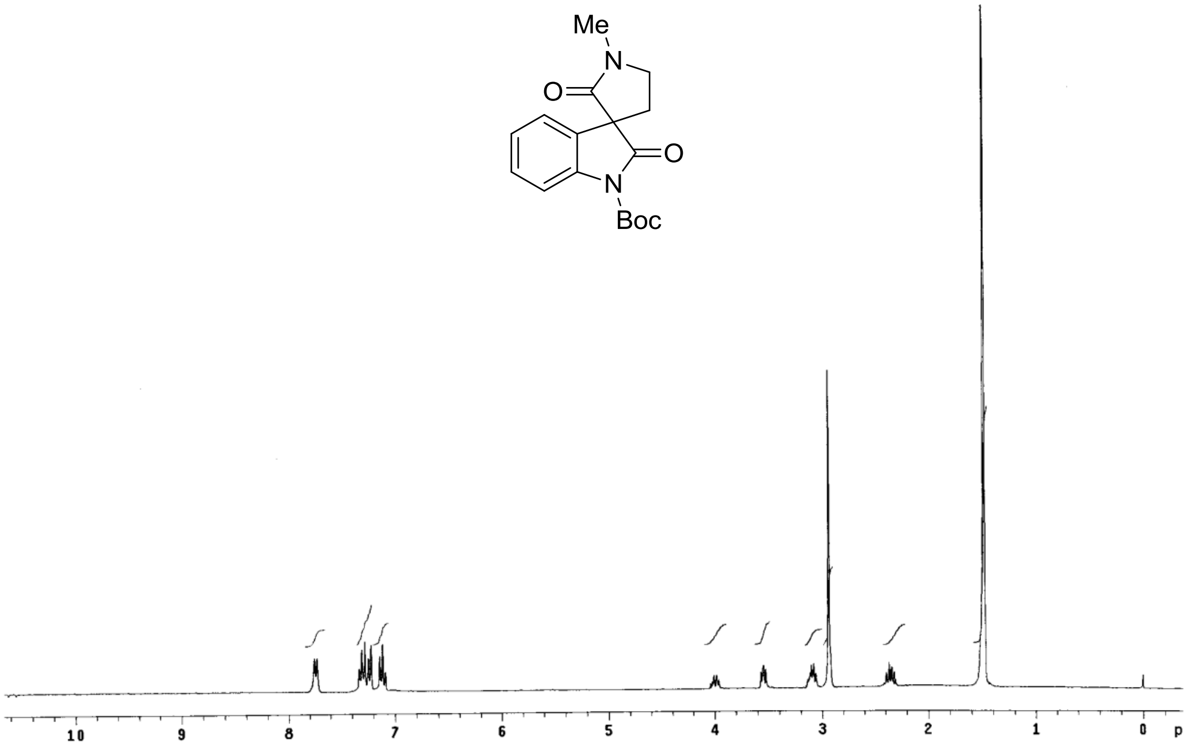

^{1}H NMR (300 MHz) Spectrum of the compound **13**

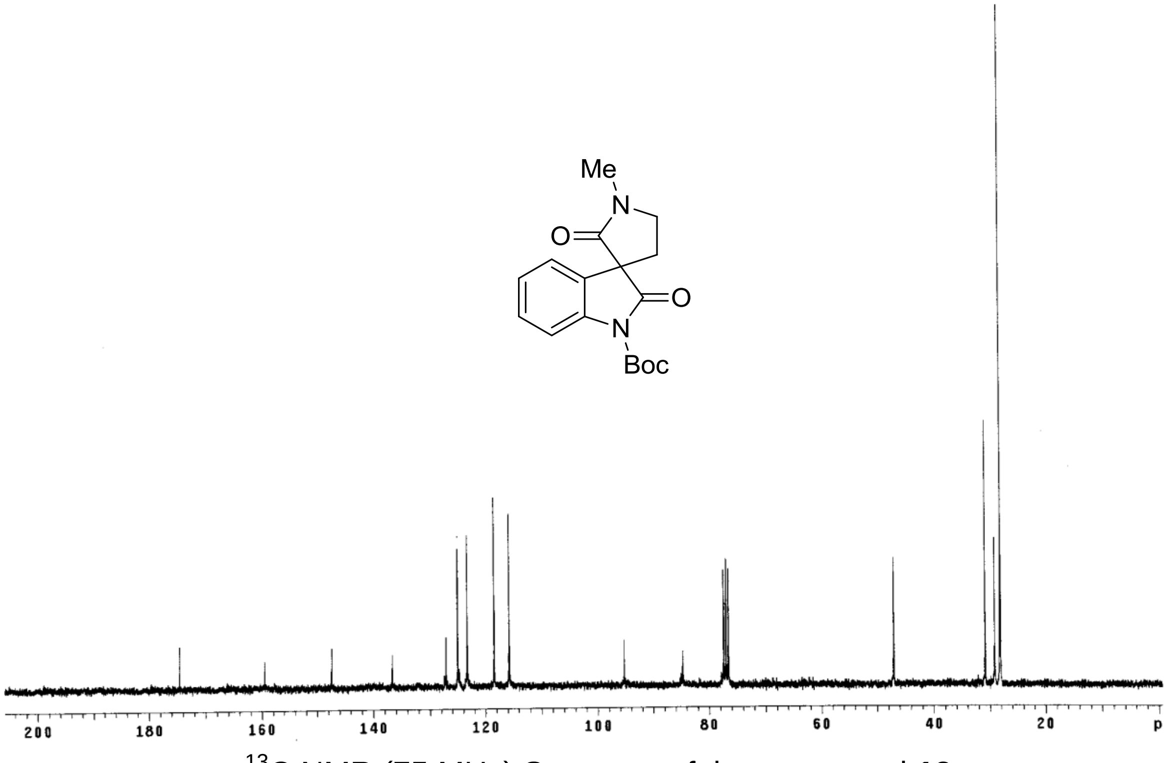

^{13}C NMR (75 MHz) Spectrum of the compound **13**

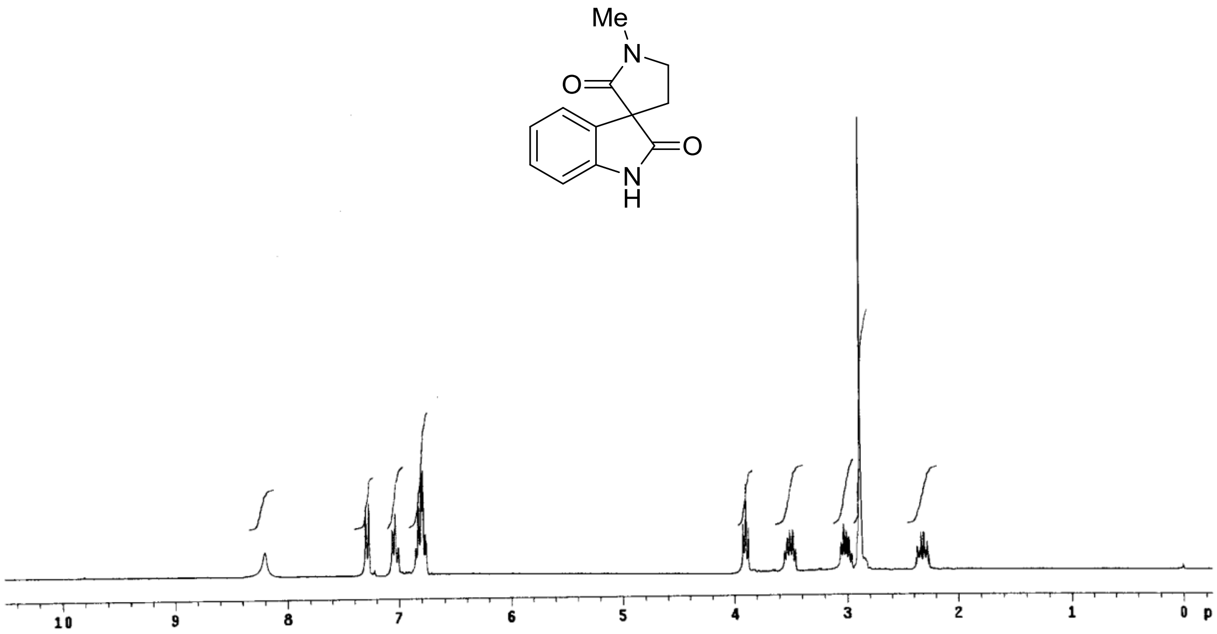

^{1}H NMR (300 MHz) Spectrum of the compound **14**

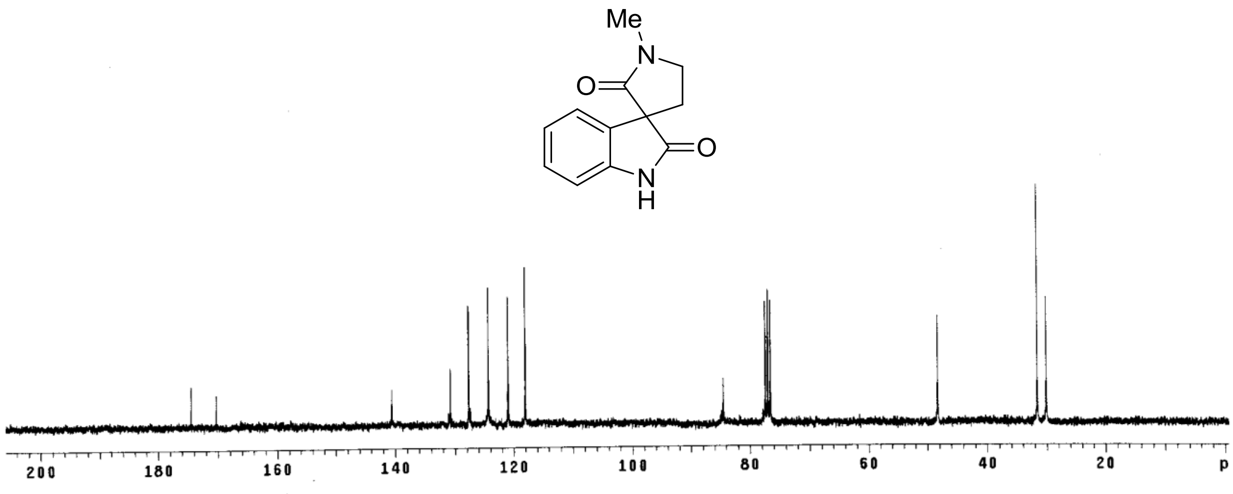

^{13}C NMR (75 MHz) Spectrum of the compound **14**

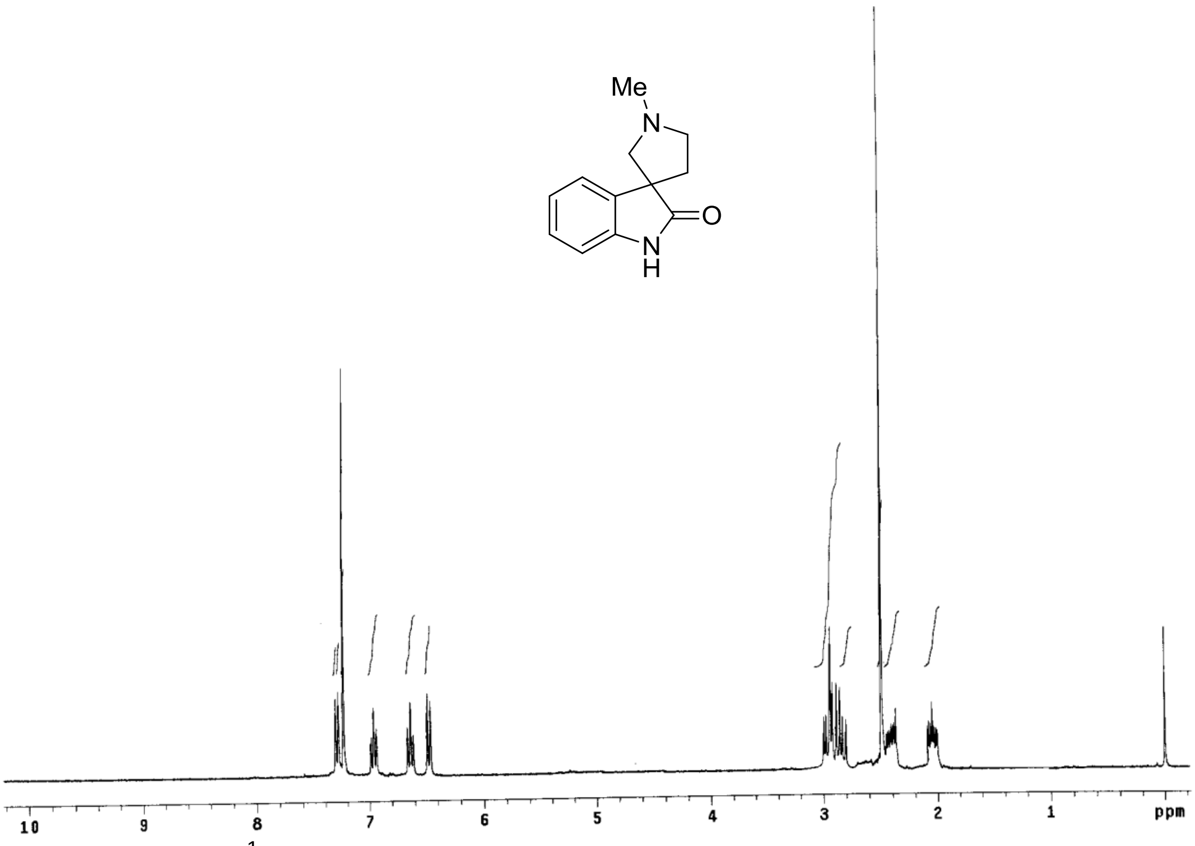

^{1}H NMR (300 MHz) Spectrum of the compound **1**

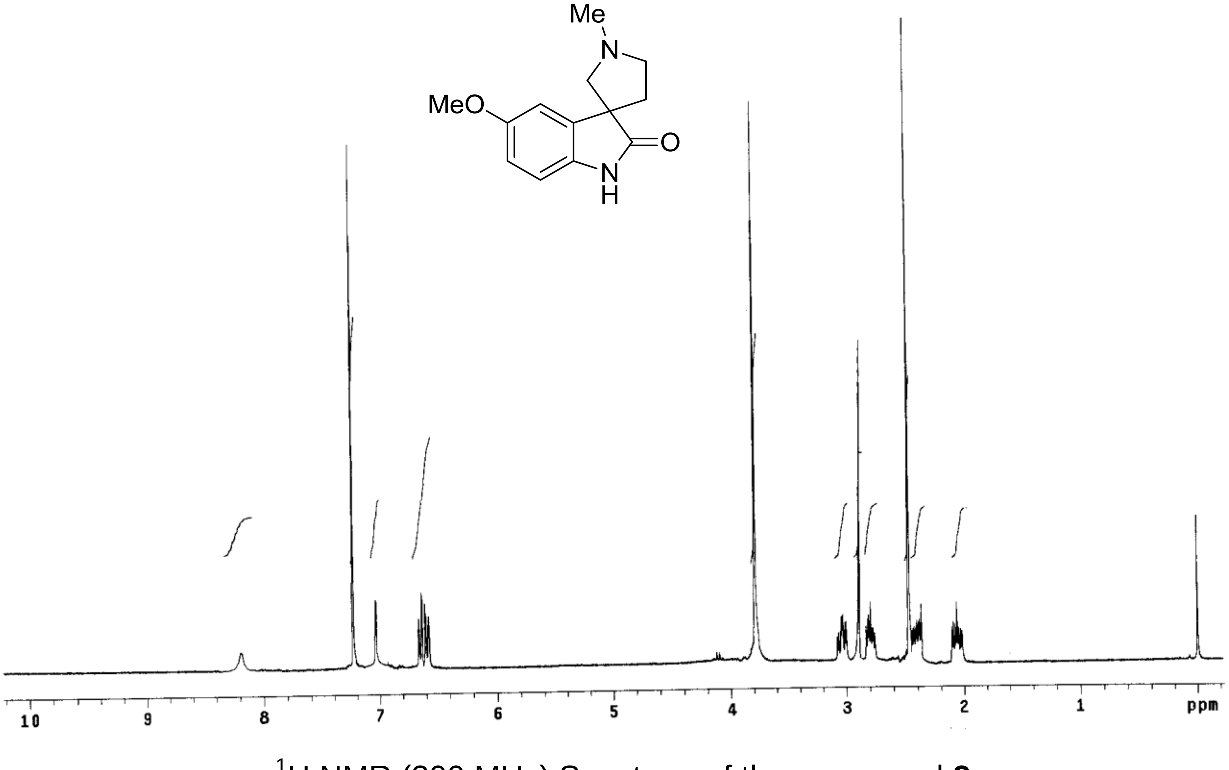

^{1}H NMR (300 MHz) Spectrum of the compound **2**
